# Development and validation of a prognostic nomogram model in locally advanced NSCLC based on metabolic features of PET/CT and hematological inflammatory indicators

**DOI:** 10.1186/s40658-024-00626-2

**Published:** 2024-03-05

**Authors:** Congjie Wang, Jian Fang, Tingshu Jiang, Shanliang Hu, Ping Wang, Xiuli Liu, Shenchun Zou, Jun Yang

**Affiliations:** 1 Department of Pulmonary and Critical Care Medicine, Yantai Yuhuangding Hospital, Yantai, Shandong China; 2 Department of thoracic surgery, Yantai Yuhuangding Hospital, Yantai, Shandong China; 3 Department of Radiation Oncology, Yantai Yuhuangding Hospital, Yantai, Shandong China; 4 Department of Radiology, Yantai Yuhuangding Hospital, Yantai, Shandong China; 5 Department of Oncology, Yantai Yuhuangding Hospital, No.20 Yuhuangding East Road, Yantai, 250117 Shandong China

**Keywords:** LA-NSCLC, Nomogram, Concurrent chemoradiotherapy, SII, SUVmean, TLG

## Abstract

**Background:**

We combined the metabolic features of ^18^F-FDG-PET/CT and hematological inflammatory indicators to establish a predictive model of the outcomes of patients with locally advanced non-small cell lung cancer (LA-NSCLC) receiving concurrent chemoradiotherapy.

**Results:**

A predictive nomogram was developed based on sex, CEA, systemic immune-inflammation index (SII), mean SUV (SUVmean), and total lesion glycolysis (TLG). The nomogram presents nice discrimination that yielded an AUC of 0.76 (95% confidence interval: 0.66–0.86) to predict 1-year PFS, with a sensitivity of 63.6%, a specificity of 83.3%, a positive predictive value of 83.7%, and a negative predictive value of 62.9% in the training set. The calibration curves and DCA suggested that the nomogram had good calibration and fit, as well as promising clinical effectiveness in the training set. In addition, survival analysis indicated that patients in the low-risk group had a significantly longer mPFS than those in the high-risk group (16.8 months versus 8.4 months, *P* < 0.001). Those results were supported by the results in the internal and external test sets.

**Conclusions:**

The newly constructed predictive nomogram model presented promising discrimination, calibration, and clinical applicability and can be used as an individualized prognostic tool to facilitate precision treatment in clinical practice.

**Supplementary Information:**

The online version contains supplementary material available at 10.1186/s40658-024-00626-2.

## Introduction

Non-small cell lung cancer (NSCLC) is the most prevalent form of lung cancer, accounting for approximately 85% of lung cancer cases worldwide [1]. Due to its insidious nature, nearly one-third of patients have progressed to locally advanced non-small cell lung cancer (LA-NSCLC) by the time they are diagnosed and have lost the optimal opportunity for surgery [2]. Concurrent chemoradiotherapy (CCRT) is the standard of therapy for inoperable LA-NSCLC and has been established since the 1990s [3]. However, most patients still experience recurrence, and the 5-year survival rate is only 15–25% [4, 5]. The use of biomarkers for the early prediction of patient prognosis may help clinicians to perform risk stratification and adjust the intensity and type of therapy for individualized management.

^18^F-deoxyglucose positron emission tomography/computed tomography (^18^F-FDG PET/CT) provides quantitative information on the metabolic activity of tumors, which is utilized not only for the diagnosis, staging, and assessment of the effectiveness of cancer treatments but also for the prediction of patients outcomes [6–8]. The metabolic features of PET demonstrated to yield more valuable prognostic information beyond conventional imaging in NSCLC [7–10]. Standardized uptake value (SUV), which represents the ratio of the radioactive activity of the imaging agent absorbed by local tissues to the average injected activity of the entire body, is a frequently employed semi-quantitative indication in PET. The most frequently used features for evaluating tumor metabolic activity in PET/CT imaging are the maximum of SUV (SUVmax), mean of SUV (SUVmean), metabolic tumor volume (MTV), and total lesion glycolysis (TLG) [9, 10]. SUVmax reflects the metabolic activity of the highest uptake site of ^18^F-FDG in tumors, while SUVmean reflects the average metabolic activity of the uptake area of FDG. MTV and TLG are different classes of metabolic features derived from the SUV that take into account the tumor volume for PET/CT imaging. MTV assesses both tumor volume and metabolic activity, which differs from the tumor volume calculated using anatomical images. TLG combines tumor metabolic volume and the metabolic uptake of FDG, providing a comprehensive measure of the overall tumor burden in the body [8, 10, 11]. However, PET/CT is susceptible to the effects of examination noise and pixel size [12–14]. In addition, the patient’s body mass index, blood glucose, and postinjection imaging time may also interfere with the screening of SUV [12–15]; therefore, there is a need to combine other indices to more objectively reflect the metabolic status of the tumor.

Inflammation is considered to be a hallmark feature in the development and progression of tumors [16, 17]. Systemic inflammatory hematological indicators based on circulating blood counts have recently been widely investigated as prognostic markers for tumors [18]. There is increasing evidence that the presence and severity of the systemic inflammatory response are intimately related to the prognosis of patients, and some inflammatory-related hematological indicators have been identified as independent predictors of patient outcomes [19–22]. The neutrophil-to-lymphocyte count ratio (NLR) [19], an early indicator of systemic inflammation, has been demonstrated to have dramatic prognostic value in a variety of malignancies, including non-small cell lung cancer. In addition, the lymphocyte-to-monocyte ratio (LMR) [20], platelet-to-lymphocyte count ratio (PLR) [21], and systemic immune-inflammation index (SII) [22] were also revealed to have similar predictive roles. However, most of these studies evaluated only blood markers alone and did not consider them in combination with other indicators.

Combining the glucose metabolism features of primary tumors from FDG-PET/CT with systemic hematological inflammatory factors, taking into account both tumor and host dimensions, may be able to improve the prognostic power for patients with LA-NSCLC while increasing the richness of information. Hence, we aimed to establish and validate a simple-to-use and effective early prediction model [23] for PFS in patients with inoperable LA-NSCLC based on clinical characteristics, metabolic features from FDG-PET/CT, and hematological inflammatory indicators. Furthermore, we investigated the prognostic differences between patients in the high- and low-risk groups based on the predictive models.

## Methods

### Patients and data collection

We retrospectively collected 149 inoperable LA-NSCLC patients who were admitted to Shandong Cancer Hospital from February 2014 to September 2017. These 149 patients were randomly split into training/validation and internal test sets at a ratio of 7:3. In addition, we retrospectively enrolled an additional 35 patients who attended Yantai Yuhuangding Hospital from January 2018 to December 2020 as an independent external test set. In the study, the training set is also a validation set but we will refer to it as a training set for simplicity. All of the patients were inoperable, LA-NSCLC based on the 7th (for the training and internal test sets) and 8th (for the external test set) editions of the AJCC staging system. We extracted the following basic characteristics from the hospital’s electronic medical record system: age, sex, smoking history, tumor location, clinical TNM stage, pathology type, CEA, NSE, and Cyfra21-1. This study was approved by the ethics committee of Yantai Yuhuangding Hospital. Additionally, because the study was retrospective in nature, informed consent was not needed.

### Treatment protocols and follow-up

All patients were treated with concurrent chemoradiotherapy. Radiotherapy techniques included three-dimensional conformal radiotherapy (3D-CRT) and intensity-modulated radiotherapy (IMRT). Chemotherapy was conducted with a cisplatin/docetaxel or a cisplatin/pemetrexed regimen at the same time as the initial radiotherapy on Day 1. Chemotherapy was cycled every 21 days for two cycles with radiation and then another 2–4 cycles without radiation. Follow-ups were undertaken every three months during the initial two years after completion of all treatments, every six months for the following three years, and then once a year thereafter. In the present study, we adopted progression-free survival (PFS) as the prognostic endpoint, which was defined as the period between the start of treatment and the objective progression of the disease or death from any cause.

### PET/CT imaging data

All patients underwent a whole-body PET/CT scan using a PET/CT scanner (Discovery LS, GE Healthcare) one week before initiating antitumor therapy. The Xeleris™ workstation (GE Healthcare) presents CT images, attenuation-corrected PET images, and merged PET/CT images as sagittal, coronal, and transverse slices. The primary tumor SUV values were determined based on a region of interest (ROI) obtained thresholding each PET image using a generally accepted threshold of 2.5. A volumetric zone of interest was sketched around the primary tumor contour on the transverse plane of the PET/CT image using semiautomatic software. To prevent overlap with nearby structures that were FDG avid, the ROI boundaries were modified by visual inspection of the original tumor. The software automatically calculated the SUVmean value and MTV. The TLG was artificially determined by multiplying the SUVmean by MTV. SUVmax, SUVmean, MTV, and TLG obtained from PET/CT were used for the analysis.

### Hematology data

Peripheral venous blood samples were taken within one week of starting the anticancer treatment. The peripheral LMR, NLR, PLR, and SII were computed based on the following equations: LMR = lymphocyte number/monocyte number; NLR = neutrophil number/lymphocyte number; PLR = platelet number/lymphocyte number; SII = neutrophil number × platelet number/lymphocyte number.

### Prognostic factors selection and nomogram development

Univariate Cox regression was utilized to screen potential PFS-related predictors in the training set. Subsequently, factors with *p*-value less than 0.05 were included in the least absolute shrinkage and selection operator (LASSO) regression to further filter more significant predictors for PFS. The LASSO method has the capability to avoid model overfitting and is suitable for regression analysis of high-dimensional data with multiple covariates. The potential predictors were selected utilizing a parameter known as the minimum mean square error criterion lambda (λ). Then, a predictive nomogram model for PFS was constructed based on the factors filtered by LASSO. This nomogram model was used to predicting PFS for specific time points (1 and 2 years). However, in univariate regression analysis, we used PFS and whether recurrence was the outcome event.

### Performance of the nomogram

Bootstrapping validation (1000 bootstrap resamples) was used to assess the nomogram model’s prediction ability in training set. The discriminative power of the nomogram was measured using receiver operating characteristic (ROC) curves and the corresponding area under the curve (AUC). To evaluate the nomogram’s identification and calibration, calibration curves were constructed. The Hosmer–Lemeshow test was performed to estimate the goodness-of-fit of the nomogram. To examine the nomogram model’s clinical applicability and overall benefit, decision curve analysis (DCA) was adopted.

### Risk group stratification based on the nomogram

The risk scores for PFS were calculated for each patient based on the nomogram, and the patients were then divided into high- and low-risk cohorts according to the optimal cutoff value obtained by the Youden index from the ROC analysis with the X-tile 3.6.1 software (Yale University, USA) in the training set. Differences in PFS between patients in the high- and low-risk cohorts were assessed in the training, internal test, and external test sets.

### Statistical analysis

The Mann‒Whitney U test was employed to compare continuous variables, and Pearson’s chi-square test was utilized to compare categorical variables. Categorical variables were described with percentages and continuous data using medians [interquartile ranges (IQRs)]. PFS was estimated utilizing the Kaplan‒Meier method, and the differences between the high-risk and low-risk groups were compared by using the log-rank test. Statistical analysis was performed with the SPSS program (V22.0, Inc., Chicago, IL, USA) and R project (4.1.3, “glmnetwere” packages for LASSO logistic regression analysis, “forestplot” packages for plotting forests, “hmisc” packages for plotting nomograms, “calibration curves” packages for plotting calibration curves, “pROC” packages for plotting ROC curves and calculating AUCs, and “stdca” packages for DCA). A two-sided *p*-value less than 0.05was regarded as statistically significant.

## Results

### Patient characteristics

**Fig. 1:**
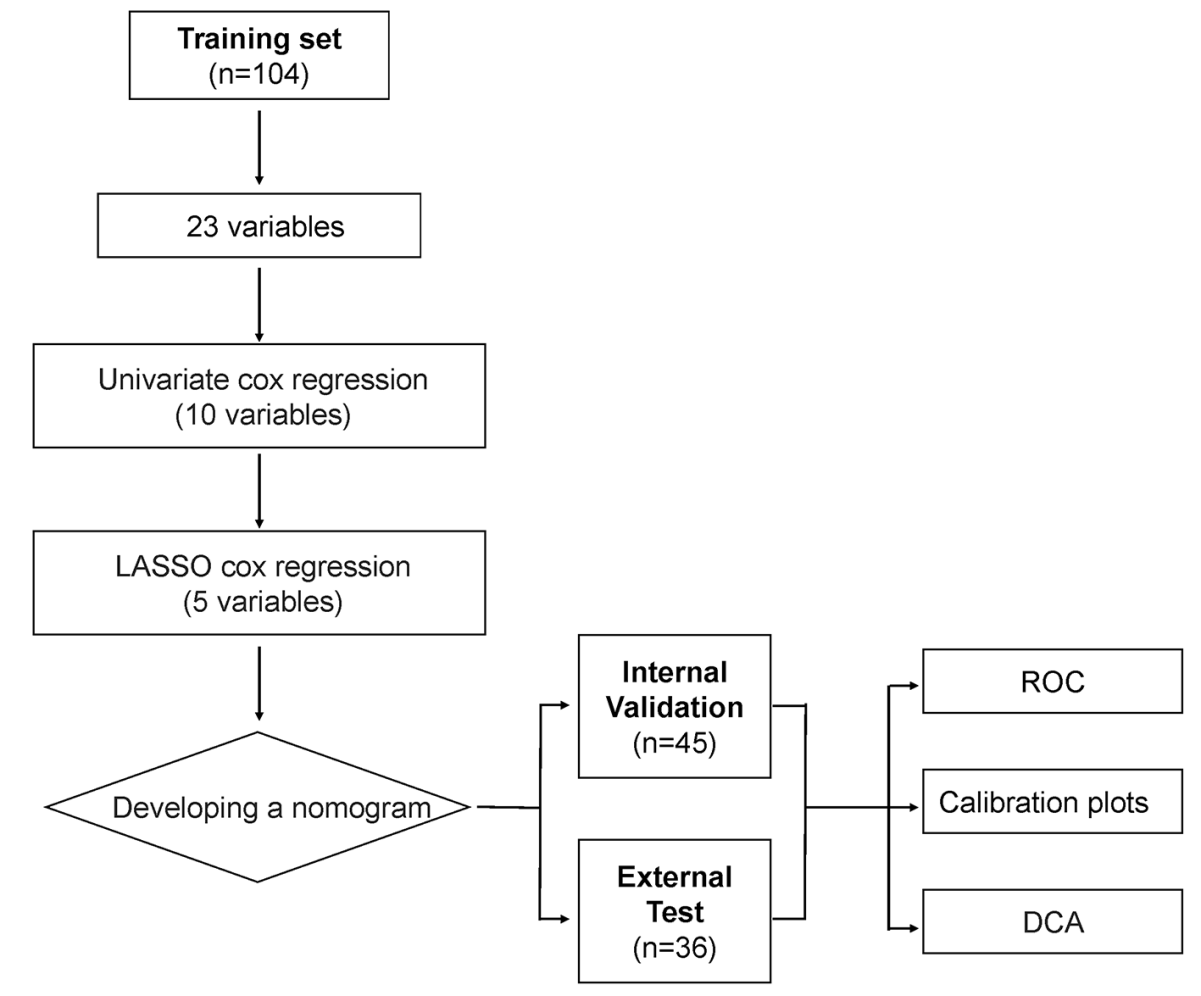
The flowchart of the study procedure

Figure 1 shows the study’s conceptual framework. There were 104, 45, and 35 patients in the training set, internal test set, and external test set, respectively. Patients in the training and internal validation sets had similar baseline characteristics (Table 1). The mPFS of the training, internal test, and external test sets were 9.43 months (95% CI: 5.7–13.2 months), 11.3 months (95% CI: 9.3–13.2 months), and 11.3 months (95% CI: 7.4–15.2 months), respectively.

**Table 1:** Baseline characteristics of patients

Variables	Training set *n* = 104	Internal test set *n* = 45	*P* ^*^	External test set *n* = 35
Age [years]			0.673	
Median (range)	61 (36–84)	60 (37–73)		65 (43–84)
Gender			0.273	
Male	72(69.2%)	27(60.0%)		26 (74.29%)
Female	32(30.8%)	18(40.0%)		9 (25.71%)
Smoking			0.759	
Yes	56(53.8%)	23(51.1%)		24 (68.57%)
No	48(46.2%)	22(48.9%)		11 (31.43%)
Tumor location			0.135	
Central	41(39.4%)	12(26.7%)		12 (34.29%)
Peripheral	63(60.6%)	33(73.3%)		23 (65.71%)
T stage			0.296	
1	20(19.2%)	7(15.6%)		6 (17.14%)
2	34(32.7%)	15(33.3%)		10 (28.57%)
3	15(14.4%)	12(26.7%)		7 (20.00%)
4	35(33.7%)	11(24.4%)		12 (34.29%)
N stage			0.954	
0	6(5.8%)	3(6.7%)		3 (8.57%)
1	3(2.9%)	2(4.4%)		6 (17.14%)
2	48(46.1%)	21(46.7%)		12 (34.29%)
3	47(45.2%)	19(42.2%)		14 (40.00%)
cTNM stage			0.254	
IIIA	36(34.6%)	13(28.9%)		18 (51.43%)
IIIB	68(46.4%)	32(71.1%)		10 (28.57%)
IIIC				7 (20.00%)
Pathology			0.307	
ADC	58 (55.8%)	21 (46.7%)		13 (37.14%)
SCC	46 (44.2%)	24 (53.3%)		22 (62.86%)
CEA [ng/ml]	11.66 [4.58, 39.4]	11.07 [4.77, 22.69]	0.755	6.05 [4.18, 11.21]
NSE [ng/ml]	14.83 [11.92, 18.41]	15.11 [12.32, 18.42]	0.711	18.92 [14.50, 21.94]
Cyfra21-1 [ng/ml]	4.04 [2.97, 6.76]	4.18 [3.02, 6.33]	0.962	5.25 [3.82, 13.52]
Lymphocyte [10^9^ cells/L]	1.74 [1.28, 2.13]	1.78 [1.48, 2.23]	0.430	1.67 [1.35, 2.39]
Neutrophil [10^9^ cells/L]	5.12 [3.95, 6.07]	4.71 [4.06, 6.00]	0.761	4.82 [3.18, 6.50]
Monocyte [10^9^ cells/L]	0.51 [0.40, 0.69]	0.54 [0.41, 0.72]	0.315	0.54 [0.40, 0.82]
Platelet [10^9^ cells/L]	269.00 [218.00, 328.00]	263.00 [191.00, 326.00]	0.872	258.00 [210.00, 302.00]
LMR	3.51 [2.40, 4.55]	3.17 [2.39, 4.34]	0.564	3.18 [2.05, 4.61]
NLR	2.86 [2.17, 3.86]	2.96 [2.01, 3.39]	0.699	2.73 [1.83, 4.06]
PLR	159.07 [110.70, 215.19]	144.74 [110.39, 186.49]	0.377	151.83 [105.09, 191.55]
SII	764.81 [532.54, 1088.50]	753.56 [454.07, 1108.47]	0.421	721.08 [417.97, 1067.48]
SUVmax	12.57 [8.71, 15.82]	11.64 [8.45, 15.22]	0.581	11.47 [9.65, 14.66]
SUVmean	5.08 [4.18, 6.32]	4.66 [3.90, 5.80]	0.076	5.92 [4.17, 6.96]
MTV	40.90 [14.98, 74.37]	26.82 [11.28, 59.01]	0.113	42.97 [17.15, 82.78]
TLG	210.00 [68.72, 437.71]	126.61 [45.01, 320.18]	0.085	272.54 [94.95, 673.87]

*: Comparison of clinical characteristics between training, internal test, and external test sets

Abbreviation: ADC: adenocarcinoma; SCC: squamous cell carcinoma; LMR: lymphocyte to monocyte ratio; NLR: neutrophil to lymphocyte ratio; PLR: platelet to lymphocyte ratio; SII: systemic immune-inflammation index; MTV: metabolic tumor volume; TLG: tumor lesion glycolysis

### Factor selection and construction of the nomogram

**Fig. 2:**
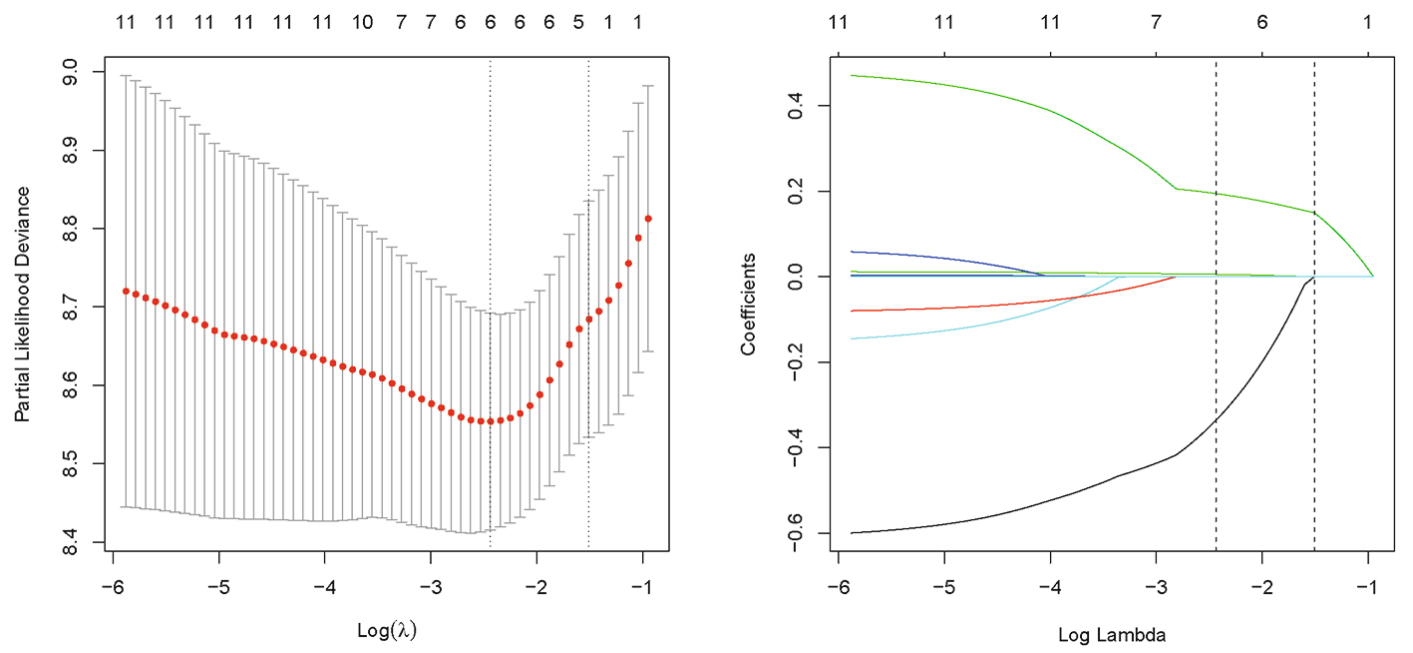
Feature selection using the least absolute shrinkage and selection operator (LASSO) Cox regression model. LASSO coefficient profiles of the 10 features. Selection of tuning parameter (λ) in the LASSO regression using 10-fold cross-validation via minimum criteria. At the optimal values log (λ), where features are selected, two dotted vertical lines were drawn at the optimal scores by minimum criteria and 1-s.e. criteria (**A**), Coefficient profile plot was produced against the log(λ) sequence. (**B**)

**Fig. 3:**
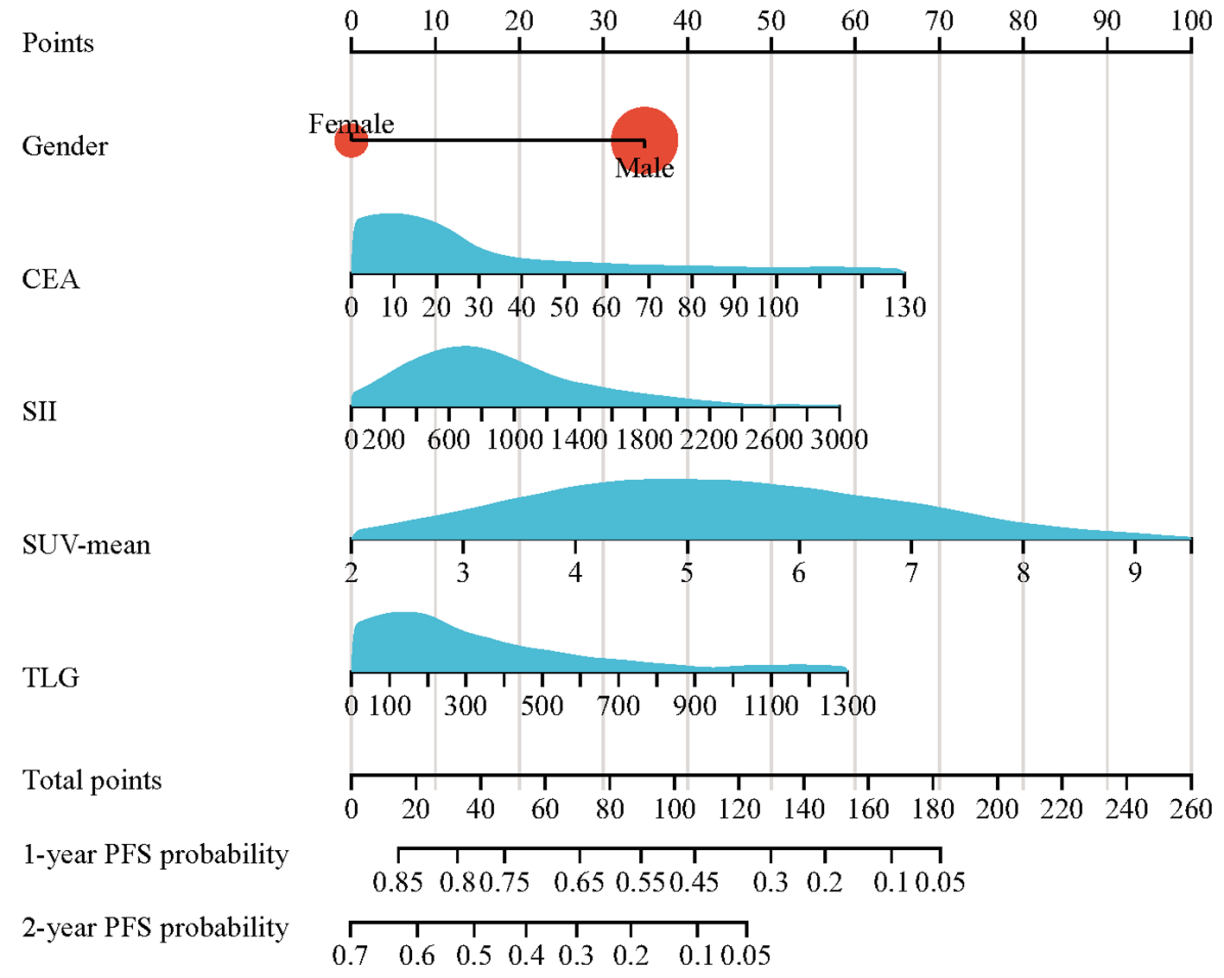
The nomogram was developed in the training set. It included five factors: gender, CEA, systemic immune-inflammation index (SII), SUVmean, and total lesion glycolysis (TLG). The nomogram plot provides a visual way to predict the PFS of patients. By drawing a vertical line from the total points axis to the risk axis, the probability of 1-year PFS and 2-year PFS for the patient could be estimated

Univariate Cox regression analysis for the training set suggested that 10 of the 23 factors had a significant correlation with PFS (Table 2). They were sex (*P* = 0.028), CEA (*P* = 0.004), neutrophils (*P* = 0.013), NLR (*P* = 0.009), PLR (*P* = 0.027), SII (*P* = 0.001), SUVmax (*P* = 0.008), SUVmean (*P* < 0.001), MTV (*P* = 0.002), and TLG (*P* < 0.001). Then, feature selection was performed by LASSO logistic regression analysis of the ten variables. The results show that the optimal value of tuning parameter λ in the LASSO logistic regression was 0.087 when the mean-squared error reached its minimum value. Five variables with nonzero coefficients were screened: sex, CEA, SII, SUVmean, and TLG (Fig. 2). Finally, a predictive nomogram model for PFS was constructed (Fig. 3). Based on this nomogram, the point scale scores for these five variables could be calculated for each patient, and their sum was the total point value.

**Table 2:** Univariate cox regression model for PFS in training set

Variables	HR	95%CI	*P*
Age	0.993	0.974–1.013	0.493
Gender (male vs. female)	1.697	1.060–2.716	0.028
Smoking (Yes vs. No)	1.418	0.930–2.162	0.105
Tumor location (peripheral vs. central)	0.794	0.521–1.210	0.283
T stage			
1	-		
2	0.786	0.432–1.430	0.430
3	0.896	0.430–1.867	0.770
4	1.221	0.681–2.188	0.502
N stage			
0	-		
1	0.318	0.059–1.717	0.183
2	0.774	0.303–1.973	0.591
3	0.831	0.327–2.112	0.698
cTNM stage			
IIIA	-		
IIIB	1.079	0.665–1.750	0.759
IIIC	1.639	0.909–2.956	0.101
Pathology (ADC vs. SCC)	0.718	0.472–1.090	0.120
CEA	1.009	1.003–1.016	0.004
NSE	1.013	0.987–1.040	0.322
Cyfra21-1	1.025	0.995–1.056	0.100
Lymphocyte	0.715	0.496–1.031	0.073
Neutrophil	1.181	1.035–1.348	0.013
Monocyte	1.797	0.681–4.742	0.237
Platelet	1.002	1.000-1.005	0.112
LMR	0.993	0.880–1.122	0.914
NLR	1.148	1.035–1.273	0.009
PLR	1.002	1.000-1.005	0.027
SII	1.001	1.000-1.001	0.001
SUVmax	1.050	1.013–1.088	0.008
SUVmean	1.406	1.215–1.627	< 0.001
MTV	1.006	1.002–1.010	0.002
TLG	1.002	1.001–1.002	< 0.001

Abbreviation: CI: confidence interval; ADC: adenocarcinoma; SCC: squamous cell carcinoma; LMR: lymphocyte to monocyte ratio; NLR: neutrophil to lymphocyte ratio; PLR: platelet to lymphocyte ratio; SII: systemic immune-inflammation index; MTV: metabolic tumor volume; TLG: tumor lesion glycolysis

### Performance evaluation

**Fig. 4:**
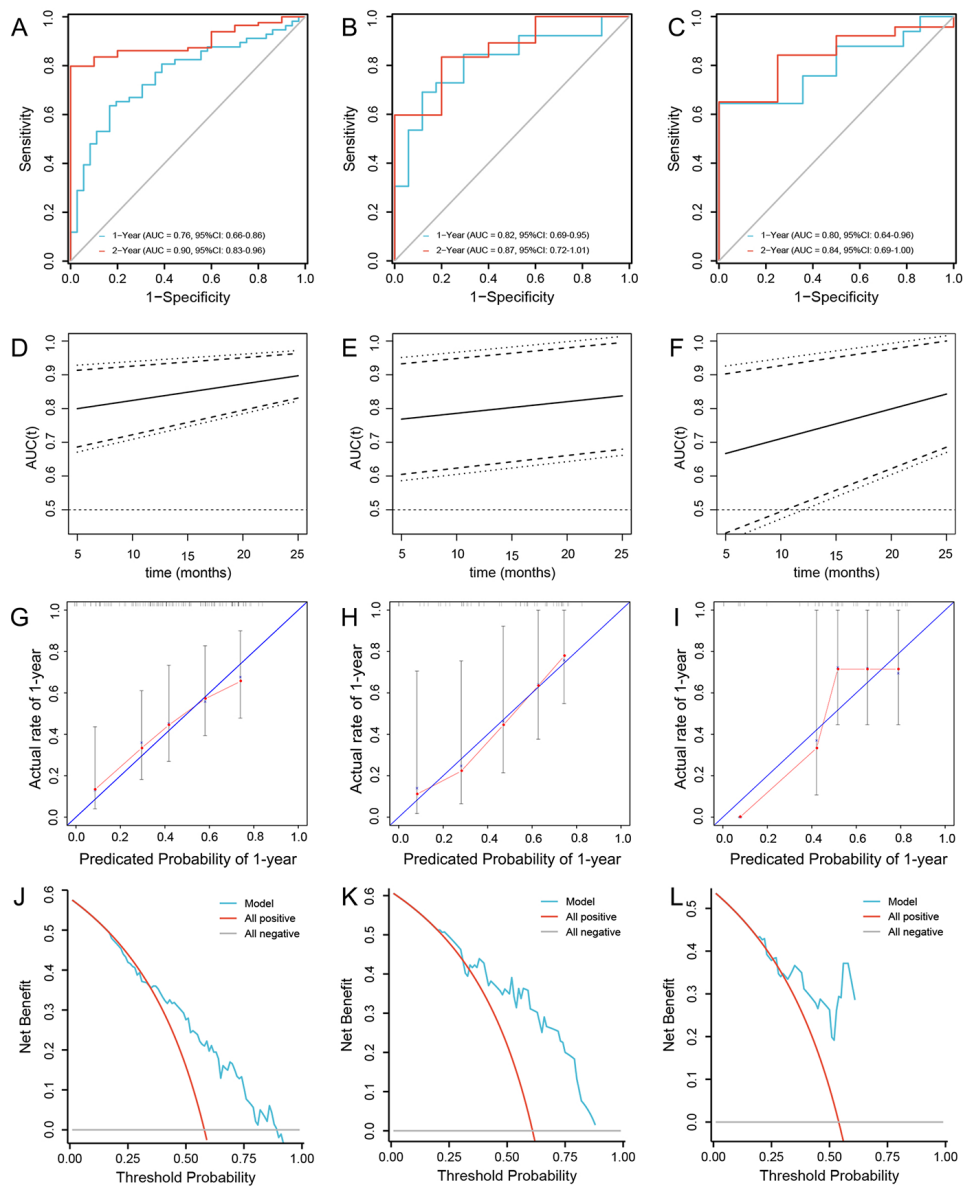
Evaluation of the performance of the nomogram. Validation of the discrimination power of the nomogram by ROC curve analysis in the training (**A**) internal test (**B**) and external test set (**C**); Time-dependent AUCs indicated that the nomogram had favorable accuracy for predicting PFS in the range of 5 months to 25 months (**D**, **E**, and **F**). Calibration plot of the nomogram in the training (**G**) internal test (**H**) and external test set (**I**), the blue diagonal line indicates the perfect prediction of the ideal model. The solid black line represents the performance of the nomogram, and the closer the fit to the diagonal line, the more accurate the prediction. The gray dashed line represents the performance of the model trained after bootstrapping validation (1000 bootstrap resamples), which corrects the overfitting situation; DCA analysis of the nomogram in the training (**J**) internal test (**K**) and external test set (**L**). The Y-axis represents the net benefit, the X-axis represents the threshold probability. The red line represents the nomogram, and the blue and orange lines represent the all-patient treatment scenario and the no-patient treatment scenario, respectively. Abbreviations: ROC: receiver operating characteristic; AUC: area under the curve; DCA: decision curve analysis

The performance of the nomogram model was evaluated in the training set. The ROC curve showed that the nomogram had favorable discrimination for PFS, with an AUC of 0.76 (95% CI: 0.66–0.86), a sensitivity of 63.6%, a specificity of 83.3%, a positive predictive value (PPV) of 83.7%, and a negative predictive value (NPV) of 62.9% for 1-year PFS; and an AUC of 0.90 (95% CI: 0.83–0.96), a sensitivity of 79.8%, a specificity of 100.0%, a PPV of 100.0%, and an NPV of 44.1% for 2-year PFS (Fig. 4A). Time-dependent AUCs indicated that the nomogram had favorable accuracy for predicting PFS in the range of 5 months to 25 months (Fig. 4D). The calibration curves visually revealed favorable accordance between the prediction of the nomogram and the actual observations (Fig. 4G). The Hosmer–Lemeshow test demonstrated a nice goodness-of-fit of the nomogram, with no significant differences observed (*P* = 0.247). DCA showed that the nomogram had a nice overall net benefit in the threshold probability range of 35.0–89.0% (Fig. 4J), indicating that the model has promising clinical effectiveness. These results suggest that the nomogram has excellent performance in the training set. The model’s accuracy was then assessed using an internal test set and an external test set. Consistent with the results in the training set, the nomogram yielded a favorable AUC of 0.80 (95% CI: 0.64–0.96) with a sensitivity of 64.4%, a specificity of 100.0%, a PPV of 100.0%, and an NPV of 71.8% for 1-year PFS; and an AUC of 0.84 (95% CI: 0.69–0.99), a sensitivity of 65.0%, a specificity of 100.0%, a PPV of 100.0%, and an NPV of 32.9% for 2-year PFS in the internal test set (Fig. 4B, Supplementary Fig. 1B, E); an AUC of 0.82 (95% CI: 0.69–0.95), a sensitivity of 69.1%, a specificity of 88.2%, a PPV of 89.8%, and an NPV of 65.58% for 1-year PFS; and an AUC of 0.87 (95% CI: 0.72-1.00), a sensitivity of 83.4%, a specificity of 80.0%, a PPV of 96.7%, and an NPV of 41.19% for 2-year PFS in external test set (Fig. 4C, Supplementary Fig. 1C, F). The time-dependent AUCs also indicated that the model had superior accuracy in both the internal test set and the external test set (Fig. 4C, F). The calibration curve and Hosmer–Lemeshow test suggested that the nomogram had good calibration and fit in both the internal and external test sets (Fig. 4E, I). Moreover, DCA visually showed that the nomogram had an overall net benefit within a wider threshold probability in the internal test set (Fig. 4K, L; Supplementary Fig. 2B, C). Besides, we performed additional analysis to compare the predictive efficacy of the nomogram with that of a single indicator. The AUC of the nomogram was better than that of the single factors of SII, SUVmean, and TLG, with AUCs of 0.696, 0.702, 0.70, and 0.681 for 1-year PFS and AUCs of 0.770, 0.848, and 0.782 for 2-year PFS, respectively in the training set (Supplementary Fig. 1A, D). DCA showed that the nomogram was superior to the single factors of SII, SUVmean, and TLG in the training set (Supplementary Fig. 2A), These results suggest that the nomogram functions well and has excellent predictive capability.

### Performance of the nomogram in the risk stratification of patients

**Fig. 5:**
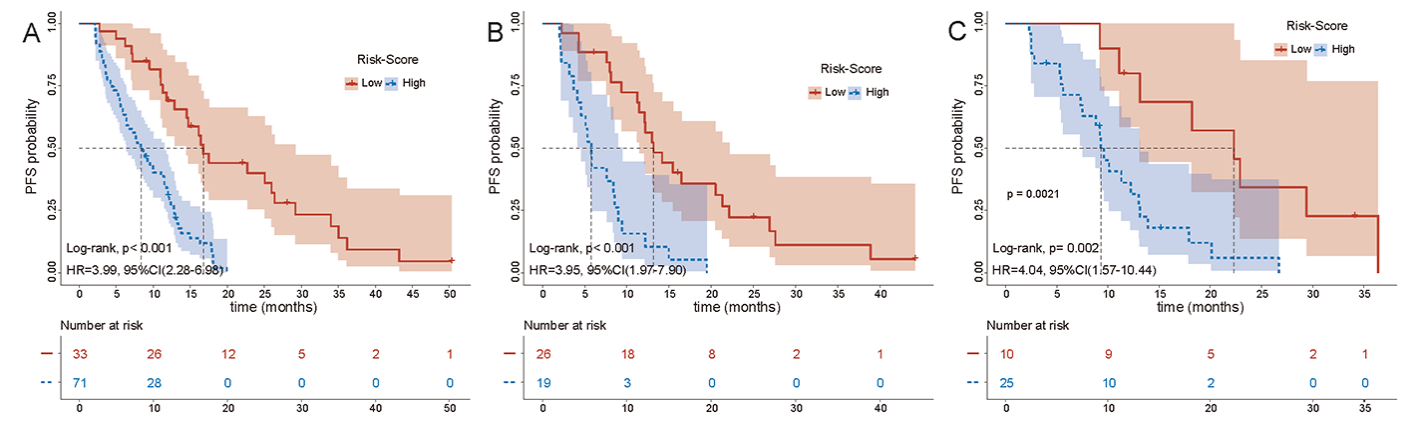
Kaplan-Meier curves for PFS of high-risk patients and low-risk patients in the training (**A**) internal test (**B**) and external test set (**C**). The PFS of patients in the high-risk group was significantly shorter than that of low-risk patients

The three sets of patients were classified into high- and low-risk groups using the optimal cutoff value of 0.165 obtained from the ROC analysis in the training set. The mPFS for patients in the low-risk group (*n* = 33) was 16.8 months (95% CI: 12.9–26.0 months), which was significantly longer than that of the 8.4 months (95% CI: 6.3–11.1 months) for patients in the high-risk group (*n* = 71) (HR: 0.25, 95% CI: 0.14–0.44, *P* < 0.001) (Fig. 5A). Similarly, in the internal test set, we also observed a significantly longer mPFS in the low-risk group than in the high-risk group (mPFS: 13.2 versus 5.8 months, HR: 0.25, 95% CI: 0.13–0.51, *P* < 0.001) (Fig. 5B). This was supported by the results of the external test set, where the mPFS was 22.3 months and 9.3 months for the low- and high-risk groups, respectively, with statistically significant differences (HR: 0.25, 95% CI: 0.10–0.64, *P* < 0.002) (Fig. 5C). These results indicate that the nomogram model can be applied to predict the PFS of patients with LA-NSCLC.

## Discussion

In the present study, we constructed a nomogram model based on the metabolic features of PET/CT and the inflammatory indicators of the peripheral blood to predict the PFS of patients with inoperable LA-NSCLC who received concurrent chemoradiotherapy. The model had promising discrimination, calibration, and clinical applicability. To the best of our knowledge, this is the first prognostic model for patients with LA-NSCLC that integrates the two dimensions of tumor metabolism and host immune inflammation. The model can provide valuable information to clinicians for the early identification of subgroups of populations of LA-NSCLC with a poor prognosis following CCRT. This will effectively optimize the treatment strategy at an early stage and provide patients with appropriate personalized care and intervention management, thus enabling individualized and precise treatment and an improved prognosis.

The metabolic-volume parameter of ^18^F-FDG PET/CT has been demonstrated to be an independent prognostic factor in various kinds of tumors. Using PET/CT as a potential prognostic indicator has also attracted extensive attention from scholars [9–11]. SUVmax presents information only about individual volume pixels within the tumor but does not evaluate the volume or heterogeneity of the metabolically active lesions. In contrast to it, SUVmean is the mean value of SUV within the sketched ROI, which reflects the mean uptake of ^18^F-FDG within the ROI and represents a superior picture of the metabolic activity of the tumor [24]. TLG, on the other hand, reflects the total glycolytic rate of the active tumor tissue. It indirectly indicates the active degree of tumor cells and has been suggested to have an advantage over the other metabolic features of PET/CT in the prognostic assessment of patients [25–27]. In the study by Moon et al., 234 patients with stage IV lung adenocarcinoma who underwent PET/CT before chemotherapy were analyzed. The multivariate Cox proportional risk regression model showed that TLG was a significant independent predictor of the PFS and OS of patients [25]. For patients with advanced NSCLC who did not undergo surgery, Yıldırım et al. [26] analyzed 110 patients with advanced (stage IIIa-IV) NSCLC, all of whom received CCRT after PET/CT. A multifactorial Cox proportional risk regression model revealed that only low TLG (< 225.7) was an independent predictor of the OS of patients. The above study suggested that the greater the tumor burden is, the higher the total metabolic rate and the shorter the time of cell multiplication, all of which indicated that the prognosis of the patients is relatively poor.

SII is a newly developed systemic immune inflammatory index that uses neutrophil, lymphocyte, and platelet counts to quantify systemic inflammation. Compared to a single or a combination of two indicators [22, 28], SII provides a more comprehensive picture of the host’s immune status and inflammatory response, and its predictive value may be superior to that of LMR, NLR, and PLR. SII has been demonstrated to be a novel prognostic factor for a variety of malignancies, including NSCLC [29–34]. Guo et al. [31] conducted a retrospective study of 569 patients with NSCLC who underwent surgery. The result revealed that only SII was an independent prognostic factor for OS according to the multivariate analysis. Their findings indicated that SII is a promising prognostic factor with a better predictive value than NLR and PLR for NSCLC patients who were treated with surgery. Deng et al. retrospectively analyzed 203 NSCLC patients who were treated with first-line generation EGFR tyrosine kinase inhibitors and evaluated the prognostic value of SII, NLR, and PLR. The multivariate analysis showed that NLR, PLR, and SII were independent prognostic factors for PFS, while only SII was an independent prognostic factor for OS. This finding also indicates that SII has a relatively higher prognostic value [32]. A retrospective analysis of patients with NSCLC who were treated with nivolumab revealed that pretreatment SII was an independent predictor of PFS and OS [33]. For patients with LA-NSCLC receiving concurrent radiotherapy, a retrospective study including 332 patients revealed that a high pretreatment SII was significantly associated with a low treatment response. The pretreatment SII was an important independent predictor of OS. Patients with a low SII had a significantly longer median OS than patients with a high SII (30 months versus 10 months) [34]. The predictive role of SII as a comprehensive assessment index can be illustrated by the functions of platelets, neutrophils, and lymphocytes. Platelets can promote the angiogenesis and metastasis of tumors and protect cancer cells from antitumor immune responses [35]. Neutrophils can participate in the proliferation and metastasis of tumors by releasing inflammatory mediators such as neutrophil elastase and interleukins [36, 37]. Contrary to the functions of platelets and neutrophils, tumor-associated lymphocyte infiltration is generally indicative of a good prognosis of patients, as the immune response prevents the growth and metastasis of the tumor [38].

Previous studies have investigated the relationship between the metabolic features of PET/CT and blood inflammation indicators as well as their prognostic value in malignancies [39–41]. For example, a study of patients with colorectal cancer demonstrated that NLR and LMR correlated significantly with MTV and TLG [39]. Studies of patients with head and neck cancers have also revealed a significant positive correlation between NLR and MTV and TLG [40]. A retrospective study of 132 patients with NSCLC demonstrated that there was a significant positive correlation between NLR and PLR with MTV and LTG., high NLR (≥ 6.34), PLR (≥ 291.6), MTV (≥ 79.3), and LTG (≥ 674.6) were significantly associated with a poor prognosis [41]. Based on the above theories, PET/CT reflects the functional metabolism of tumor cells and can be used to evaluate the biological behavior of tumors, while systemic inflammatory immunomarkers can indicate the balance between pro- and antitumor activity. Their combined application for predictive analysis of patient prognosis not only reflects the systemic inflammatory response status of the patients but also represents the metabolic profile of the tumors.

In this study, we constructed a predictive nomogram model for PFS based on SII, SUVmean, and TLG, which were selected by univariate regression and LASSO. No such predictive models based on a combined indicator have been reported before. The model is highly accurate and clinically adaptive compared to a single indicator. The model exhibits superior predictive performance both in the internal test set and in an independent external test set. There are other predictive models (scores or biomarkers) for the prognosis of patients with NSCLC that have been reported in previous studies. Matteo et al. [42] constructed an immune metabolic prognostic index (IMPI) for patients with NSCLC treated with nivolumab based on MTV and SII. The results showed that IMPI was significantly associated with the prognosis of patients. The mOS of patients with low, intermediate, and high IMPI was 17.5 months, 9.4 months, and 3.2 months, respectively (*p* < 0.01). In another study of 149 patients with stage III-IV NSCLC receiving chemotherapy, the researchers constructed a scoring system (SUV-LMR score) based on SUVmax and LMR. They found that the SUV-LMR score was not only significantly associated with the treatment response but was also an independent predictor of PFS and OS [43]. In addition, studies have reported the prognostic value of NLR, SII and bone marrow-to-liver SUVmax ratios (BLRs) in patients with advanced NSCLC treated with chemotherapy or immunotherapy [44]. Compared to these models, our nomogram has great advantages. In the present study, we screened indicators based on three dimensions of clinical information, PET features and blood inflammation markers to construct a predictive nomogram model. The richness of its predictors is more sophisticated. In addition to SII, SUVmean, and TLG, the sex of the patient and CEA were also utilized for the construction of the nomogram, since they have been demonstrated to correlate with the prognosis of patients. Moreover, unlike previous studies, we did not simply group patients according to the cutoff values and then assign a corresponding score, ignoring the magnitude of the contribution of factors to the prediction. Our nomogram sufficiently examines the contribution of each factor to the prognosis and grants them appropriate weights in the calculation of the risk score, this results in a more accurate and individualized prognostic risk score for patients. Importantly, we verified the performance of the nomogram in both the internal and external test sets and found that the nomogram displayed promising identification, goodness-of-fit, discriminative power, and clinical effectiveness. Overall, the combination of SII with SUVmean and TLG optimizes the performance of the nomogram model even more, which may provide a quantitative and pragmatic predictive tool for risk stratification of patients undergoing CCRT. Meanwhile, their combination expands the new perspective of integrating ^18^F-FDG PET/CT images and hematologic inflammatory indicators.

Of course, there are still some limitations of this study. First, this is not a prospective study, and there are some biases in the collection of the data of patients, such as small sample size and short observation time, which may have an impact on the stability of the results. Second, this study only used the features of pretreatment PET/CT and the pretreatment blood inflammation indicators for the construction of the model, and the above indicators may change considerably during the treatment period. Whether there are more appropriate time points for evaluation needs to be further explored. In addition, definitive evidence is lacking for the correlation between the features of PET and the inflammatory response. Therefore, basic studies, as well as prospective controlled clinical trials with larger sample sizes, are needed to validate our results.

## Conclusion

We constructed a predictive nomogram model for the PFS of patients with inoperable LA-NSCLC who received concurrent radiotherapy based on clinical information, metabolic features of PET/CT, and the inflammatory indicators of peripheral blood. The model presents promising discrimination, calibration, and clinical applicability based on the AUCs of ROC, calibration curves, and decision curve analysis. The nomogram model is helpful to reasonably and effectively optimize the allocation and utilization of medical resources at an early stage to provide appropriate care and intervention management for patients, thereby improving their prognosis.

### Electronic supplementary material

Below is the link to the electronic supplementary material.

**Supplementary Material 1: Supplementary Fig. 1** Comparation of the AUCs of the Nomogram with SII, SUVmean, and TLG for predicting 1-year PFS in the training (A) internal test (B) and external test set (C), as well as for 2-year PFS (D, E, and F). Abbreviations: AUC: area under the curve; TLG: total lesion glycolysis

**Supplementary Material 2: Supplementary Fig. 2** Comparation of the clinical applicability of the Nomogram with SII, SUVmean, and TLG by DCA in the training (A) internal test (B) and external test set (C). Abbreviations: SII: systemic immune-inflammation index; TLG: total lesion glycolysis; DCA: decision curve analysis

## Data Availability

The datasets used and/or analyzed during the current study are available from the corresponding author on reasonable request.

## Abbreviations

18 F-FDG PET/CT: 18 F-deoxyglucose positron emission tomography/computed tomography
AUC: Area under the curve
CCRT: Concurrent chemoradiotherapy
DCA: Decision curve analysis
LA-NSCLC: Locally advanced non-small cell lung cancer
LASSO: Least absolute shrinkage and selection operator
LMR: Lymphocyte-to-monocyte ratio
MTV: Metabolic tumor volume
NLR: Neutrophil-to-lymphocyte count ratio
NSCLC: Non-small cell lung cancer
PFS: Progression-free survival
PLR: Platelet-to-lymphocyte count ratio
ROC: Receiver operating characteristic
ROI: Region of interest
SII: Systemic immune-inflammation index
SUVmax: Maximum of SUV
SUVmean: Mean of SUV
TLG: Total lesion glycolysis
