# Supplementary figures and images for: Development and validation of a prognostic nomogram model in locally advanced NSCLC based on metabolic features of PET/CT and hematological inflammatory indicators
Source: EJNMMI Phys. 2024 Mar 5;11:24. doi: 10.1186/s40658-024-00626-2 (PMC10914655; doi:10.1186/s40658-024-00626-2)

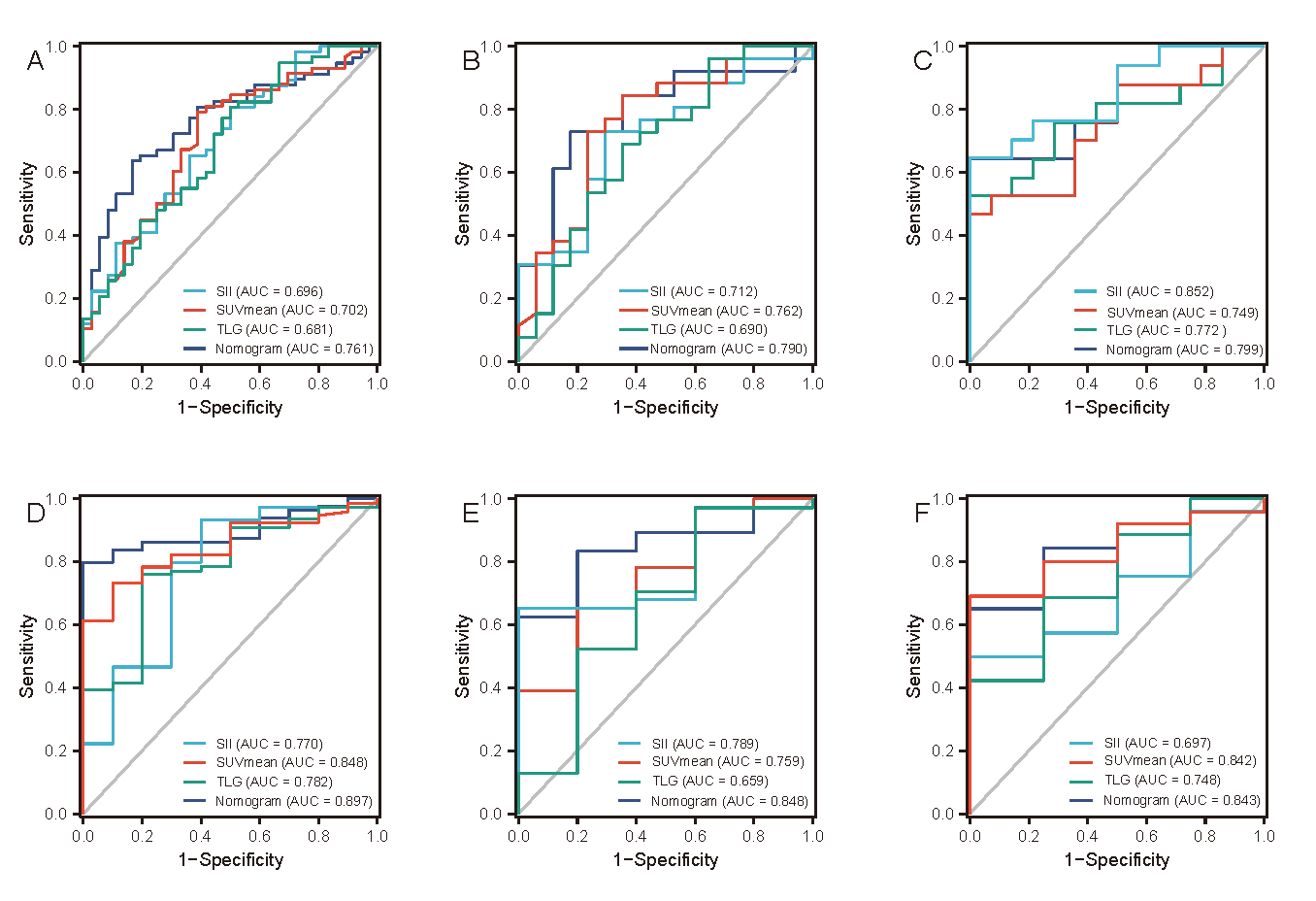

Supplement: Supplementary file 1 — Supplementary Material 1: Supplementary Fig. 1 Comparation of the AUCs of the Nomogram with SII, SUVmean, and TLG for predicting 1-year PFS in the training (A) internal test (B) and external test set (C), as well as for 2-year PFS (D, E, and F). Abbreviations: AUC: area under the curve; TLG: total lesion glycolysis [file 40658_2024_626_MOESM1_ESM.png]

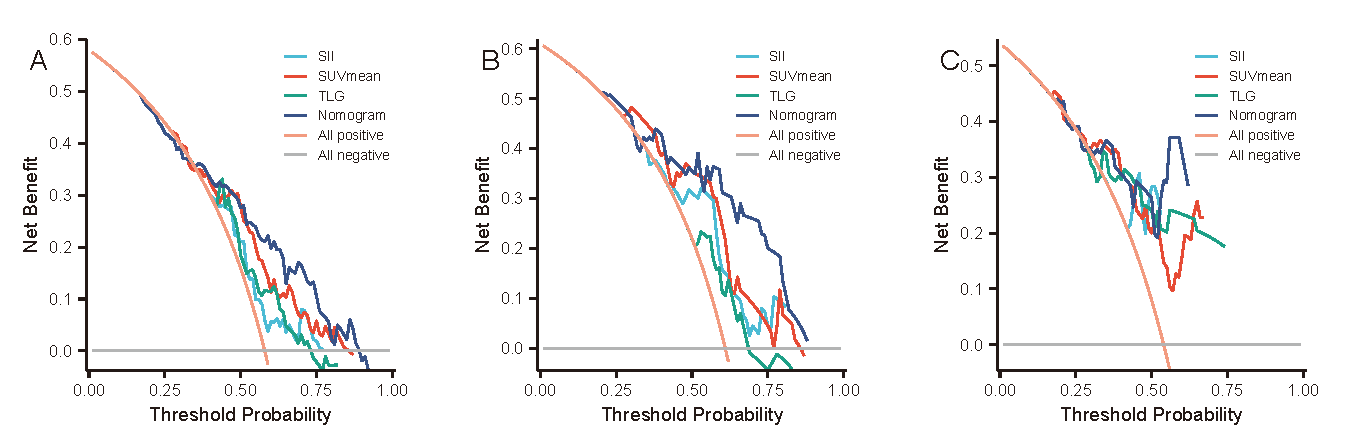

Supplement: Supplementary file 2 — Supplementary Material 2: Supplementary Fig. 2 Comparation of the clinical applicability of the Nomogram with SII, SUVmean, and TLG by DCA in the training (A) internal test (B) and external test set (C). Abbreviations: SII: systemic immune-inflammation index; TLG: total lesion glycolysis; DCA: decision curve analysis [file 40658_2024_626_MOESM2_ESM.png]
